# Supplementary material for: Toxoplasma gondii exploits the host ESCRT machinery for parasite uptake of host cytosolic proteins
Source: PLoS Pathog. 2021 Dec 13;17(12):e1010138. doi: 10.1371/journal.ppat.1010138 (PMC8700025; doi:10.1371/journal.ppat.1010138)
Supplement: S1 Fig — A. Representative images for segmentation and quantification of GFP-TSG101 and ALIX to the PVM between WT, RΔgra14 and R:GRA14OE. The PV was labeled using an antibody against TgGRA1. Scale bar is 5 μm. (DOCX) [file ppat.1010138.s001.docx]

**
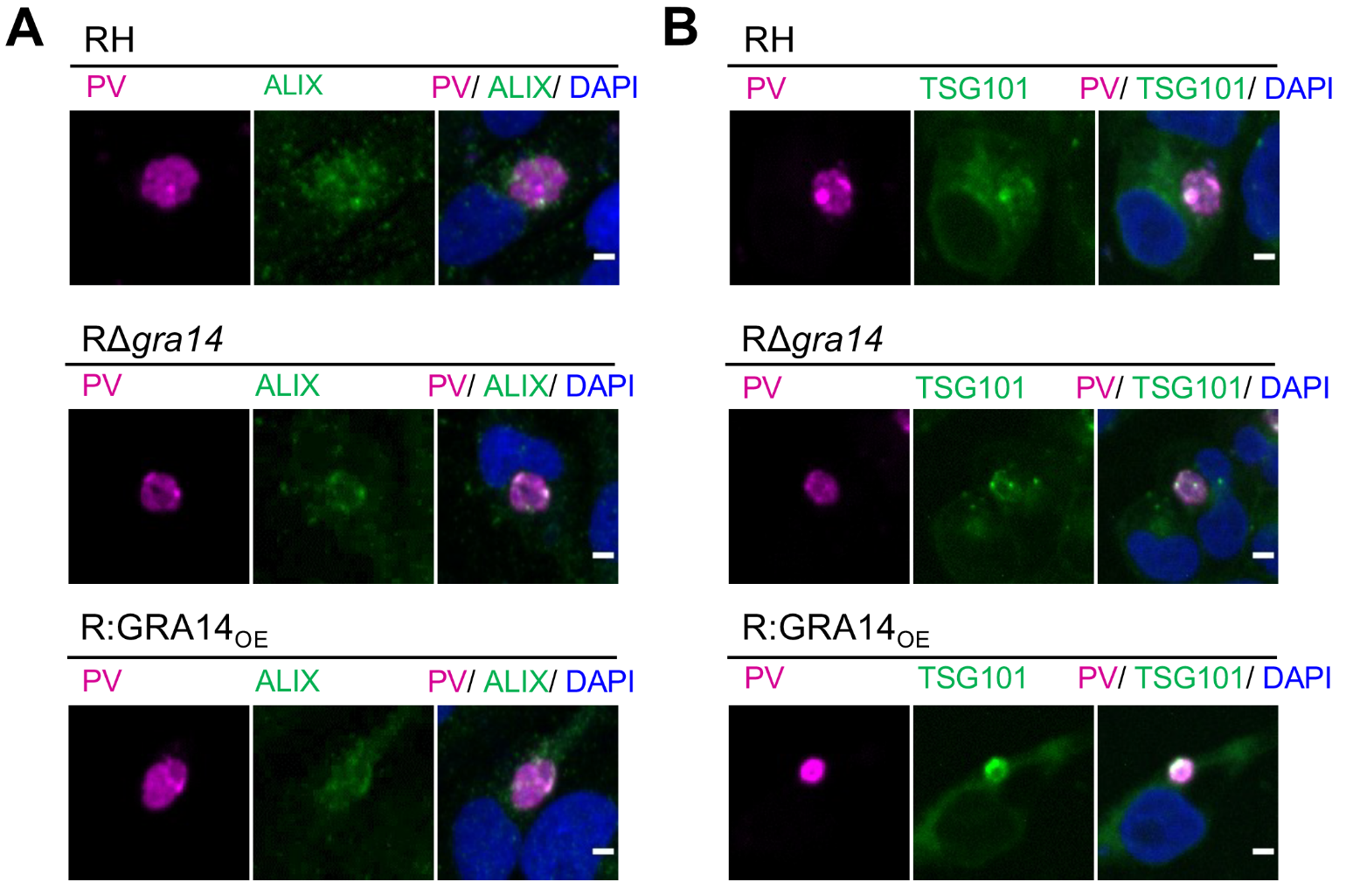
**

**S1 Fig. Comparison of ALIX and GFP-TSG101 recruitment to the PVM**

**A.** Representative images for segmentation and quantification of GFP-TSG101 and ALIX to the PVM between WT, RΔ*gra14* and R:GRA14_OE_. The PV was labeled using an antibody against TgGRA1. Scale bar is 5 µm.
