# Supplementary material for: Toxoplasma gondii exploits the host ESCRT machinery for parasite uptake of host cytosolic proteins
Source: PLoS Pathog. 2021 Dec 13;17(12):e1010138. doi: 10.1371/journal.ppat.1010138 (PMC8700025; doi:10.1371/journal.ppat.1010138)
Supplement: S2 Fig — A. Schematic for the tagging strategy with primer binding sites. B. PCR to validate the tagging of TgGRA14 with HA in the ME49Δku80 background. C. Representative images showing the co-localization of HA with TgGRA14 in the tagged strain. Scale bar is 5 μm. (DOCX) [file ppat.1010138.s002.docx]

**
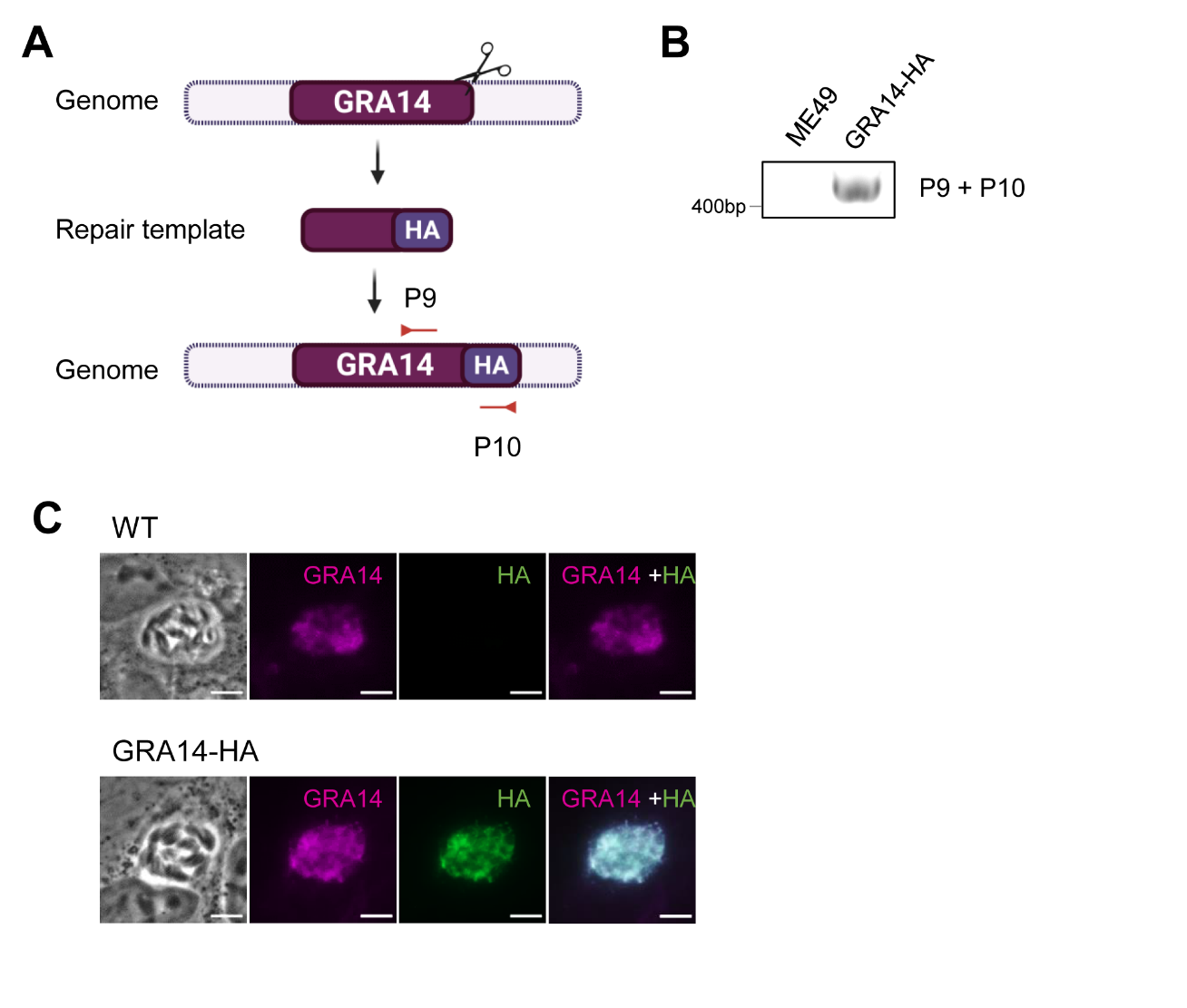
**

**S2 Fig.** **Endogenous C-terminal tagging of TgGRA14 in a type II strain**

**A.** Schematic for the tagging strategy with primer binding sites. **B.** PCR to validate the tagging of TgGRA14 with HA in the ME49Δ*ku80* background. **C.** Representative images showing the co-localization of HA with TgGRA14 in the tagged strain. Scale bar is 5 µm.
