# Supplementary material for: Toxoplasma gondii exploits the host ESCRT machinery for parasite uptake of host cytosolic proteins
Source: PLoS Pathog. 2021 Dec 13;17(12):e1010138. doi: 10.1371/journal.ppat.1010138 (PMC8700025; doi:10.1371/journal.ppat.1010138)
Supplement: S3 Fig — A. Experimental design for the analysis of the internalization of host cytosolic proteins of TgGRA14-deficient parasites. (1) Parasites were treated with 5 μM LHVS for 24 h prior to infection, (2) inducible mCherry HeLa cells were infected with parasites for 4 h, (3) the parasites were harvested at 4 hpi and analyzed by microscopy. B. Quantification of host cytosolic mCherry uptake at 4 hpi by WT or RΔgra14 type I strains treated with DMSO or LHVS for 24 h. At least 200 parasites were analyzed per blinded sample. Data represents the mean from ≥ 3 biological replicates. Statistical analysis was by Student’s t-test. Only statistical differences are shown. (DOCX) [file ppat.1010138.s003.docx]

**
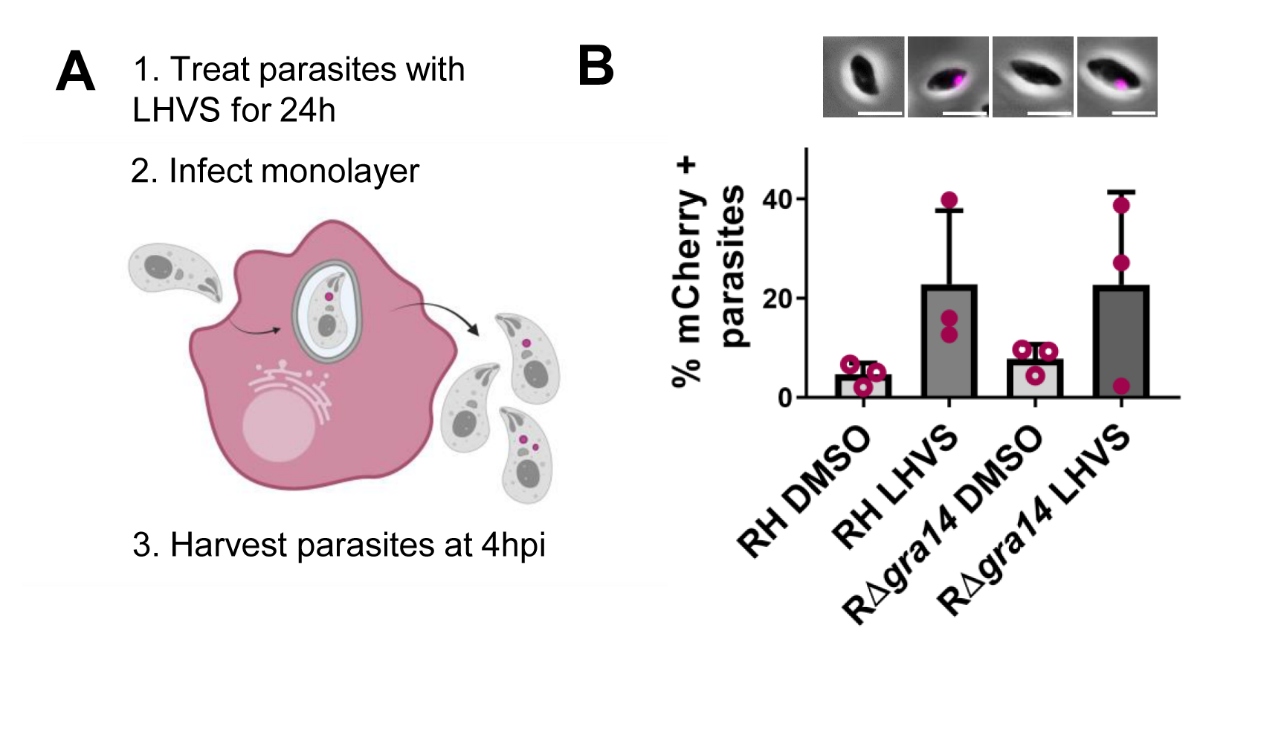
**

**S3 Fig. TgGRA14 is not needed for the internalization of host cytosolic proteins early in infection**

**A.** Experimental design for the analysis of the internalization of host cytosolic proteins of TgGRA14-deficient parasites. (1) Parasites were treated with 5 μM LHVS for 24 h prior to infection, (2) inducible mCherry HeLa cells were infected with parasites for 4 h, (3) the parasites were harvested at 4 hpi and analyzed by microscopy. **B.** Quantification of host cytosolic mCherry uptake at 4 hpi by WT or RΔ*gra14* type I strains treated with DMSO or LHVS for 24 h. At least 200 parasites were analyzed per blinded sample. Data represents the mean from ≥ 3 biological replicates. Statistical analysis was by Student’s t-test. Only statistical differences are shown.
