# Supplementary material for: Toxoplasma gondii exploits the host ESCRT machinery for parasite uptake of host cytosolic proteins
Source: PLoS Pathog. 2021 Dec 13;17(12):e1010138. doi: 10.1371/journal.ppat.1010138 (PMC8700025; doi:10.1371/journal.ppat.1010138)
Supplement: S4 Fig — A. Schematic for the deletion of the HXGPRT gene with primer binding sites B. PCR to validate the deletion of HXGPRT. C. Schematic for the deletion of the TgGRA14 with primer amplification sites. D. PCR to validate the deletion of TgGRA14 in the ME49Δku80 background. E. Immunoblot confirming the deletion of TgGRA14 in the MΔgra14 strain. (DOCX) [file ppat.1010138.s004.docx]

**
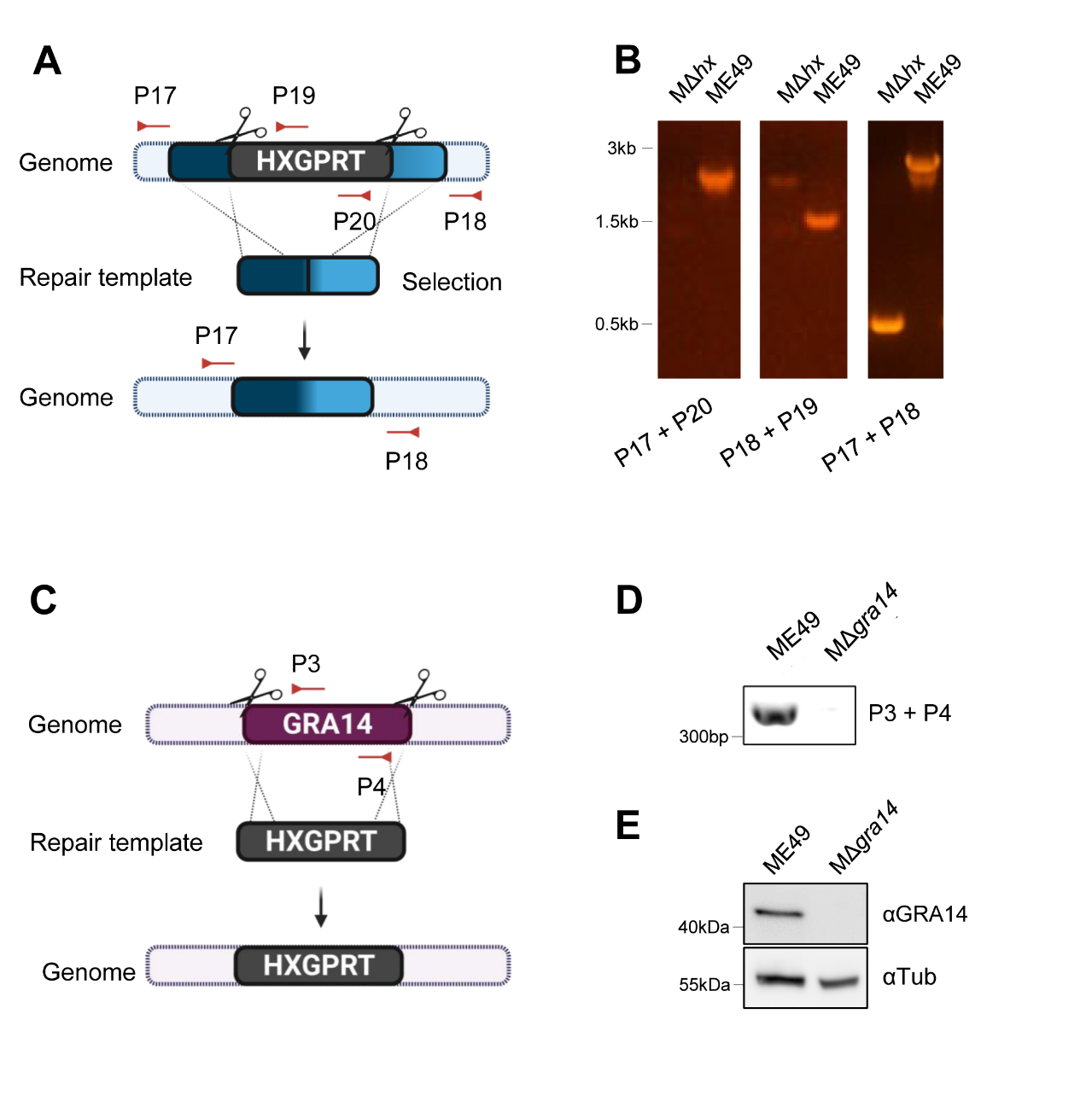
**

**S4 Fig. Deletion of TgGRA14 in type II strain**

**A.** Schematic for the deletion of the HXGPRT gene with primer binding sites **B.** PCR to validate the deletion of HXGPRT. **C.** Schematic for the deletion of the TgGRA14 with primer amplification sites. **D.** PCR to validate the deletion of TgGRA14 in the ME49Δ*ku80* background. **E.** Immunoblot confirming the deletion of TgGRA14 in the MΔ*gra14* strain.
