# Supplementary material for: Toxoplasma gondii exploits the host ESCRT machinery for parasite uptake of host cytosolic proteins
Source: PLoS Pathog. 2021 Dec 13;17(12):e1010138. doi: 10.1371/journal.ppat.1010138 (PMC8700025; doi:10.1371/journal.ppat.1010138)
Supplement: S5 Fig — A. Schematic representation of the plasmids generated for TgGRA14 complementation. B. PCR to validate the presence of TgGRA14-HA in the RΔgra14GRA14 complementation mutants. C. Immunoblot confirming the presence of TgGRA4 and HA in the RΔgra14GRA14 complementation mutants. D. Representative images showing the presence of HA and GRA14 in the RΔgra14GRA14 complementation mutants. Scale bar is 5 μm. (DOCX) [file ppat.1010138.s005.docx]

**
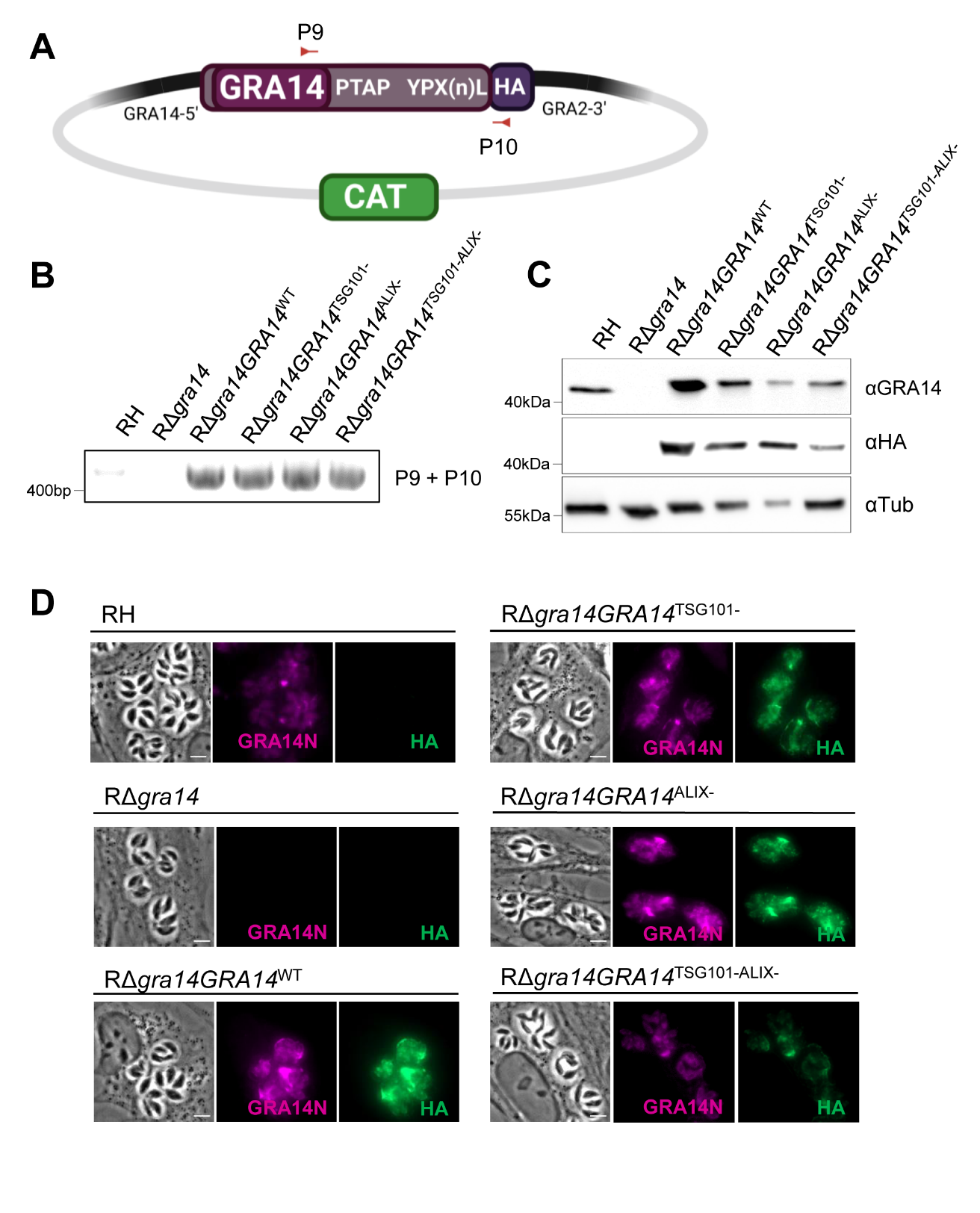
****S5 Fig. RΔ*gra14:gra14-HA* complementation mutants**

**A.** Schematic representation of the plasmids generated for TgGRA14 complementation. **B.** PCR to validate the presence of TgGRA14-HA in the RΔ*gra14GRA14* complementation mutants. **C.** Immunoblot confirming the presence of TgGRA4 and HA in the RΔ*gra14GRA14* complementation mutants. **D.** Representative images showing the presence of HA and GRA14 in the RΔ*gra14GRA14* complementation mutants. Scale bar is 5 µm.
