# Supplementary material for: Toxoplasma gondii exploits the host ESCRT machinery for parasite uptake of host cytosolic proteins
Source: PLoS Pathog. 2021 Dec 13;17(12):e1010138. doi: 10.1371/journal.ppat.1010138 (PMC8700025; doi:10.1371/journal.ppat.1010138)
Supplement: S6 Fig — A. Semi-permeabilization with 0.1% saponin for permeabilization of the host plasma membrane and PVM. B. Semi-permeabilization using 0.00001% saponin to only permeabilize the host plasma membrane and not the PVM. Detection of TgGRA14 N-terminus and C-terminus by probing against GRA14N and HA respectively. Scale bar is 5μm. (DOCX) [file ppat.1010138.s006.docx]

**
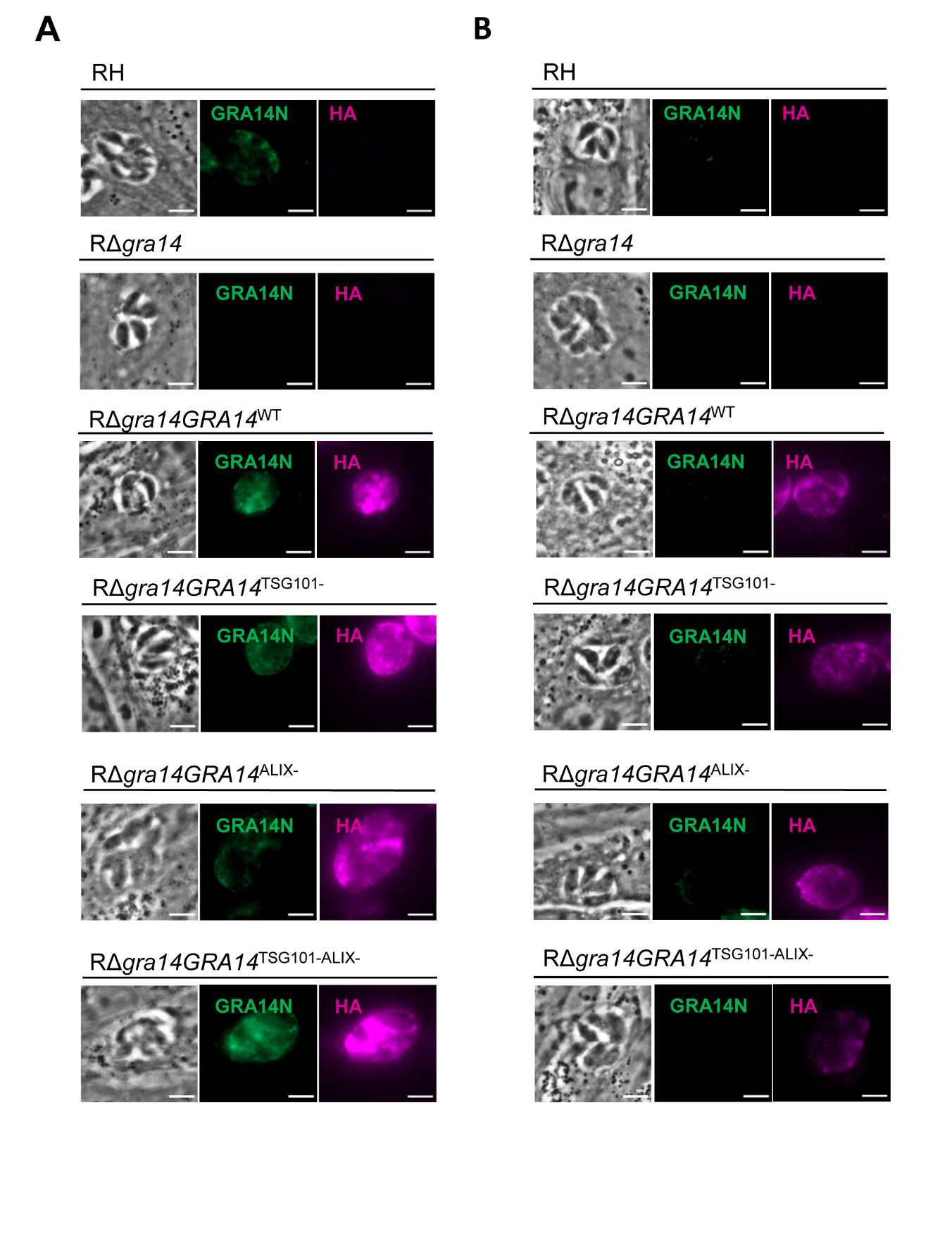
****S6 Fig. Topology of TgGRA14 late domain motif mutants at the PVM**

**A.** Semi-permeabilization with 0.1 % saponin for permeabilization of the host plasma membrane and PVM. **B.** Semi-permeabilization using 0.00001% saponin to only permeabilize the host plasma membrane and not the PVM. Detection of TgGRA14 N-terminus and C-terminus by probing against GRA14N and HA respectively. Scale bar is 5µm.
