# Supplementary material for: Toxoplasma gondii exploits the host ESCRT machinery for parasite uptake of host cytosolic proteins
Source: PLoS Pathog. 2021 Dec 13;17(12):e1010138. doi: 10.1371/journal.ppat.1010138 (PMC8700025; doi:10.1371/journal.ppat.1010138)
Supplement: S7 Fig — A. Representative images for segmentation and quantification of GFP-TSG101 to the PVM between WT, RΔgra14 and RΔgra14 complement strains. The PV was labeled using an antibody against TgGRA1. Scale bar is 5 μm. B. Replication analysis of TgGRA14 complement strains in GFP-TSG101 HeLa cells by analyzing parasites (nuclei) per PV (labeled with GRA1). C. Measurement of growth by analyzing the PV size of TgGRA14 complement strains in GFP-TSG101 HeLa cells. Data represents the mean from 3 biological replicates. Statistical analysis was by Kruskal-Wallis test. No statistical differences were detected between samples. (DOCX) [file ppat.1010138.s007.docx]

**
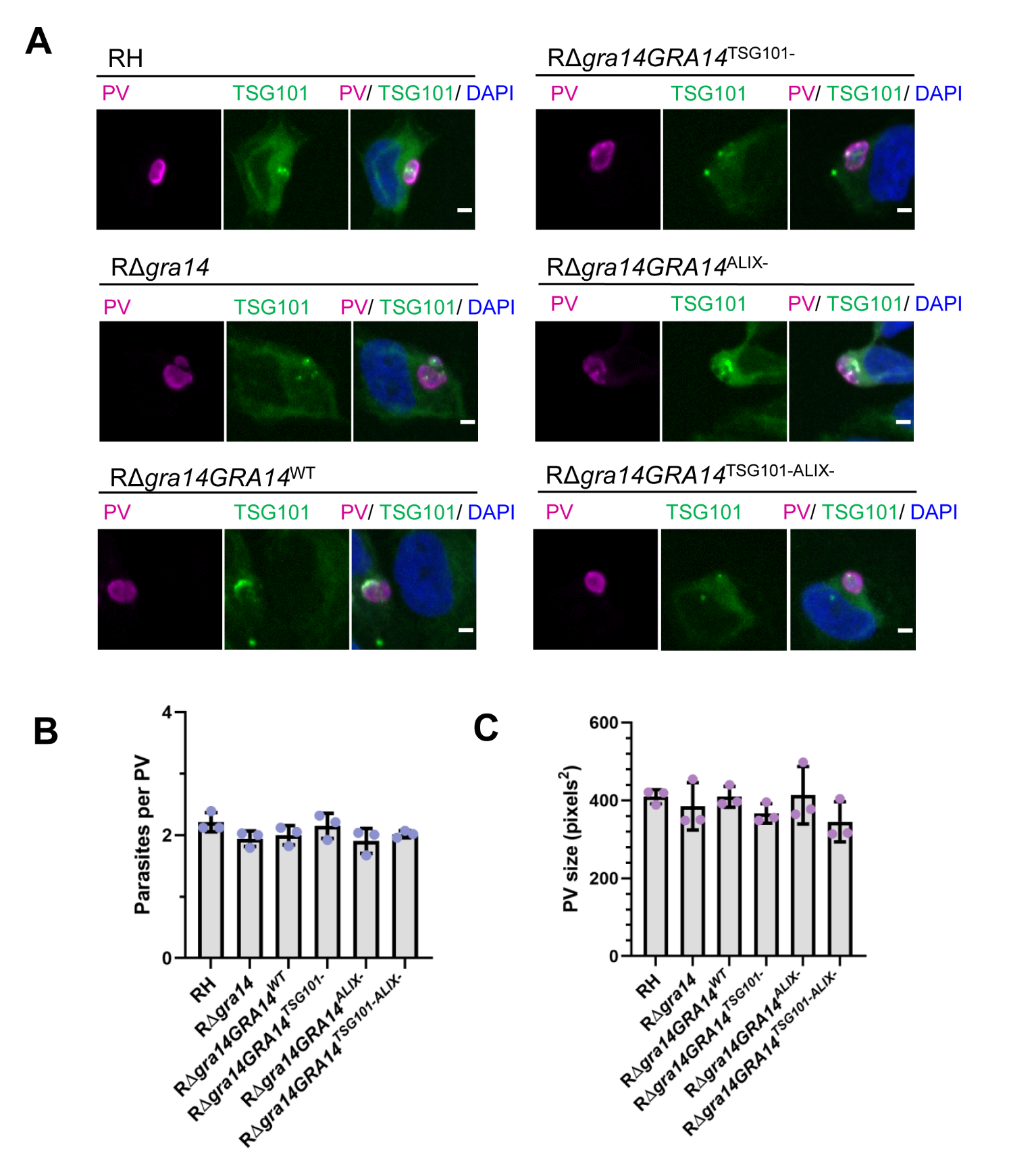
S7 Fig. Comparison of GFP-TSG101 recruitment to the PVM following disruption of late domain motifs**

**A.** Representative images for segmentation and quantification of GFP-TSG101 to the PVM between WT, RΔ*gra14* and RΔ*gra14* complement strains. The PV was labeled using an antibody against TgGRA1. Scale bar is 5 µm. **B.** Replication analysis of TgGRA14 complement strains in GFP-TSG101 HeLa cells by analyzing parasites (nuclei) per PV (labeled with GRA1). **C.** Measurement of growth by analyzing the PV size of TgGRA14 complement strains in GFP-TSG101 HeLa cells. Data represents the mean from 3 biological replicates. Statistical analysis was by Kruskal-Wallis test. No statistical differences were detected between samples.
