# Supplementary material for: Toxoplasma gondii exploits the host ESCRT machinery for parasite uptake of host cytosolic proteins
Source: PLoS Pathog. 2021 Dec 13;17(12):e1010138. doi: 10.1371/journal.ppat.1010138 (PMC8700025; doi:10.1371/journal.ppat.1010138)
Supplement: S1 Table — Identification of Toxoplasma gondii effector proteins encoding a putative P(S/T)AP motif and a signal peptide as potential candidate interactors with the host ESCRT-I component, TSG101. Bioinformatic search was performed using the vEuPathDB Toxoplasma Informatics Resources Database (ToxoDB) and their predicted localization is based on the hyperplexed localization of organelle proteins by isotype tagging (hyperLOPIT) data set [36]. (DOCX) [file ppat.1010138.s009.docx]

**S1 Table. *T. gondii* proteins encoding a P(S/T)AP motif identified bioinformatically**

| **Gene ID** | **Product Description** | **Top Predicted Location (TAGM-MCMC)** |
| --- | --- | --- |
| TGME49_239740 | Dense granule protein GRA14 | dense granules |
| TGME49_201180 | hypothetical protein | dense granules |
| TGME49_231960 | Omega secalin, putative | dense granules |
| TGME49_311470 | Rhoptry neck protein RON5 | rhoptries 1 |
| TGME49_203990 | Rhoptry protein ROP12 | rhoptries 1 |
| TGME49_312270 | Rhoptry protein ROP13 | rhoptries 1 |
| TGME49_245490 | Microneme protein MIC8 | micronemes |
| TGME49_287040 | hypothetical protein | micronemes |
| TGME49_245510 | Phospholipid-translocating P-type ATPase, flippase subfamily protein | Golgi |
| TGME49_310350 | GPI inositol-deacylase | Mitochondrion- membranes |
| TGME49_248510 | hypothetical protein | nucleolus |
| TGME49_265240 | hypothetical protein | Nucleus- chromatin |
| TGME49_201660 | hypothetical protein | null |
| TGME49_201730 | hypothetical protein | null |
| TGME49_207740 | hypothetical protein | null |
| TGME49_209620 | Peptidase A1 domain-containing protein | null |
| TGME49_211320 | hypothetical protein | null |
| TGME49_217420 | hypothetical protein | null |
| TGME49_243160 | Toxoplasma gondii family A protein | null |
| TGME49_245770 | hypothetical protein | null |
| TGME49_250670 | hypothetical protein | null |
| TGME49_254790 | hypothetical protein | null |
| TGME49_258840 | hypothetical protein | null |
| TGME49_274110 | Glycoprotease family protein | null |
| TGME49_295935 | KRUF family protein | null |
| TGME49_300320 | rRNA adenine N(6)-methyltransferase | null |
| TGME49_313120 | RPOLD domain-containing protein | null |
| TGME49_313630 | hypothetical protein | null |
| TGME49_315360 | INCENP_ARK-bind domain-containing protein | null |
| TGME49_315958 | Radical SAM domain-containing protein | null |
| TGME49_318500 | Cpw-wpc domain-containing protein | null |
| TGME49_320260 | hypothetical protein | null |
| TGME49_320540 | hypothetical protein | null |

Identification of *Toxoplasma gondii* effector proteins encoding a putative P(S/T)AP motif and a signal peptide as potential candidate interactors with the host ESCRT-I component, TSG101. Bioinformatic search was performed using the vEuPathDB *Toxoplasma* Informatics Resources Database (ToxoDB) and their predicted localization is based on the hyperplexed localization of organelle proteins by isotype tagging (hyperLOPIT) data set^34^.
