# Supplementary material for: Toxoplasma gondii exploits the host ESCRT machinery for parasite uptake of host cytosolic proteins
Source: PLoS Pathog. 2021 Dec 13;17(12):e1010138. doi: 10.1371/journal.ppat.1010138 (PMC8700025; doi:10.1371/journal.ppat.1010138)
Supplement: S2 Table — Immunoprecipitation of TgGRA14 with host proteins. List of TgGRA14 host-interacting proteins and T. gondii interacting proteins in Tz-HFF samples with >1 log2 fold change in tagged vs control lysates and a negative log2 p-value >3.32 (equivalent to p<0.1). Comparison of TgGRA14 host-interacting proteins in tachyzoite stage (Tz-HFF) with bradyzoite stage (Bz-HFF) or tachyzoites infected HFF (Tz-HFF) with tachyzoites infected neurons (Tz-Neurons). Cells are color coded with blue indicating high enrichment, white indicating moderate enrichment, and red indicating lower, but still significant, enrichment. N.D., not detected. (DOCX) [file ppat.1010138.s010.docx]

**S2 Table. Host proteins and *T. gondii* proteins significantly enriched following immunoprecipitation with TgGRA14-HA**

| **Host enriched proteins** | **Uniprot ID** |  | **Log2 Fold Change** | | |
| --- | --- | --- | --- | --- | --- |
|  |  |  | **Tz-HFF** | **Bz-HFF** | **Tz-Neuron** |
| **VPS37C** | A5D8V6 | ESCRT-I | 6.34 | 4.82 | 2.25 |
| **Vinculin** | P18206 | Actin-filament binding protein | 5.78 | 1.57 | N.D. |
| **PDCD6/ALG-2** | O75340 | Adaptor protein | 5.75 | 5.58 | 6.36 |
| **CHMP4B** | Q9H444 | ESCRT-III | 5.59 | 2.73 | 1.45 |
| **PEF1** | Q9UBV8 | ALG-2 interacting protein | 5.08 | 3.79 | N.D. |
| **CHMP4A** | Q9BY43 | ESCRT-I | 5.03 | 1.11 | N.D. |
| **UMAD1** | C9J7I0 | UBPA1-MVB12-associated (UMA) domain containing 1 | 4.73 | 3.22 | N.D. |
| **CHMP1A** | Q9HD42 | ESCRT-I | 4.50 | 1.31 | N.D. |
| **TSG101** | Q99816 | ESCRT-III | 3.86 | 2.49 | N.D. |
| **VPS37A** | Q8NEZ2 | ESCRT-I | 3.66 | 1.37 | N.D. |
| **VPS28** | Q9UK41 | ESCRT-III | 3.38 | 2.48 | 2.64 |
| **ALIX** | Q8WUM4 | Accessory protein | 2.96 | 0.94 | N.D. |
| ***T. gondii* enriched proteins** | **Uniprot ID** |  | **Log2 Fold Change** | | |
|  |  |  | **Tz-HFF** | **Bz-HFF** | **Tz-Neuron** |
| **GRA14** | TGME49_239740 |  | 5.94 | 3.44 | 4.89 |
| **GRA1** | TGME49_270250 |  | 5.86 | 6.48 | 6.95 |
| **GRA8** | TGME49_254720 |  | 5.59 | 3.45 | 4.09 |
| **GRA9** | TGME49_251540 |  | 5.26 | 4.10 | 3.91 |
| **GRA4** | TGME49_310780 |  | 5.15 | 1.45 | 3.19 |
| **MAG1** | TGME49_270240 |  | 4.92 | 3.72 | 4.47 |
| **GRA22** | TGME49_215220 |  | 4.77 | 1.73 | N.D. |
| **MYR1** | TGME49_254470 |  | 4.60 | 1.96 | 3.22 |
| **Hypothetical** | TGME49_206340 |  | 4.51 | N.D. | N.D. |
| **ERC** | TGME49_229480 |  | 4.49 | 1.95 | 2.72 |
| **GRA15** | TGME49_275470 |  | 4.47 | 0.60 | 2.13 |
| **Hypothetical** | TGME49_323100 |  | 3.79 | N.D. | N.D. |
| **GRA16** | TGME49_208830 |  | 3.72 | N.D. | N.D. |
| **GRA7** | TGME49_203310 |  | 3.59 | 3.07 | 3.82 |
| **Hypothetical** | TGME49_267740 |  | 2.99 | 0.68 | N.D. |

**S2 Table. Host proteins significantly enriched following immunoprecipitation with TgGRA14-HA**

Immunoprecipitation of TgGRA14 with host proteins. List of TgGRA14 host-interacting proteins and *T. gondii* interacting proteins in Tz-HFF samples with >1 log2 fold change in tagged vs control lysates and a negative log2 p-value >3.32 (equivalent to p<0.1). Comparison of TgGRA14 host-interacting proteins in tachyzoite stage (Tz-HFF) with bradyzoite stage (Bz-HFF) or tachyzoites infected HFF (Tz-HFF) with tachyzoites infected neurons (Tz-Neurons). Cells are color coded with blue indicating high enrichment, white indicating moderate enrichment, and red indicating lower, but still significant, enrichment. N.D., not detected.
