# Supplementary material for: Toxoplasma gondii exploits the host ESCRT machinery for parasite uptake of host cytosolic proteins
Source: PLoS Pathog. 2021 Dec 13;17(12):e1010138. doi: 10.1371/journal.ppat.1010138 (PMC8700025; doi:10.1371/journal.ppat.1010138)
Supplement: S3 Table — (DOCX) [file ppat.1010138.s011.docx]

**S3 Table. Parasite strains used in this manuscript**

| Strain | Parental strain | Genotype | Selection marker | Selection | Reference | Notes |
| --- | --- | --- | --- | --- | --- | --- |
| RH |  | RH |  |  |  |  |
| ∆*cpl* | RH |  | DHFR | Pyrimethamine |  |  |
| RH GRA14-_1X_HA  (R:GRA14_OE_) | RH∆*hpt* |  | HPT | Mycophenolic acid (MPA) + Xanthine | ^26^ | Kindly provided by Dr. P. Bradley |
| R∆*gra14* | RH∆*hpt* |  | HPT | MPA + Xanthine | ^26^ | Kindly provided by Dr. P. Bradley |
| R∆*gra14GRA14^WT^* | RH∆*gra14* | RH∆*gra14*: *gra14*^WT^*-*_1X_*HA* | CAT | Chloramphenicol | TR^1^ |  |
| R∆*gra14GRA14^TSG101-^* | RH∆*gra14* | RH∆*gra14*: *gra14*^TSG101-^*-*_1X_*HA* | CAT | Chloramphenicol | TR^1^ |  |
| R∆*gra14GRA14^ALIX-^* | RH∆*gra14* | RH∆*gra14*: *gra14*^ALIX-^*-*_1X_*HA* | CAT | Chloramphenicol | TR^1^ |  |
| R∆*gra14GRA14^TSG101-ALIX-^* | RH∆*gra14* | RH∆*gra14*: *gra14*^TSG101-ALIX-^*-*_1X_*HA* | CAT | Chloramphenicol | TR^1^ |  |
| ME49∆*ku80* | ME49 | ∆*ku80* | none | none | ^54^ |  |
| ME49 GRA14-_6X_HA | ME49∆*ku80* | ∆*ku80*∆*hx* |  |  | TR^1^ |  |
| M∆*gra14* | ME49∆*ku80* | ∆*ku80*∆*hx* | HX | MPA + Xanthine | TR^1^ |  |

^1^This report
