# Supplementary material for: Toxoplasma gondii exploits the host ESCRT machinery for parasite uptake of host cytosolic proteins
Source: PLoS Pathog. 2021 Dec 13;17(12):e1010138. doi: 10.1371/journal.ppat.1010138 (PMC8700025; doi:10.1371/journal.ppat.1010138)
Supplement: S4 Table — (DOCX) [file ppat.1010138.s012.docx]

**S4 Table. Primers used in this manuscript**

|  | Pirmer | Sequence |
| --- | --- | --- |
| P1 | **FWR GagInsert.664-1456** | CAGAAGGAGCCACCCCACAAGATTTAAATACCATGCTAAACACAGTGGGG |
| P2 | **RVS GagInsert.664-1456** | TTCCACCACCACCACCGGAAATCCCAAAATTCCCTGGCCTTCCCTTGTGG |
| P3 | **TgGRA14-Cterm.F** | ACGCCTCGTGTGCGCGCTTTTC |
| P4 | **TgGRA14-Cterm.R** | TTCGCTTGGTCTCTGGTAGCCC |
| P5 | **RVS GagInsert-GRA14Cterm.2** | CCTCTTCTCCAAAGTCTCCACTGTCAAAATTCCCTGGCCTTCCCTTGTGG |
| P6 | **FWR GRA14Cterm.2Insert.1457-1759** | CCACAAGGGAAGGCCAGGGAATTTTGACAGTGGAGACTTTGGAGAAGAGG |
| P7 | **RVS GRA14CtermInsert.1457-1759** | TTCCACCACCACCACCGGAAATCCCTTCGCTTGGTCTCTGGTAGCCCAGC |
| P8 | **GagGRA14seq.1541.fwr** | TACGTTCCGCCCATGTATCC |
| P9 | **GRA14-HA.F** | GGGGCATAAGAATGCTACAATC |
| P10 | **HA.R** | CGGGGACGTCGTACGGGTAGGC |
| P11 | **ALIXmut.Q5.F** | gctgcaCACAGGCTGGGCTACCAG |
| P12 | **ALIXmut.Q5.R** | cgcagcTAGCATAGACGCAGGGGC |
| P13 | **Q5.TSG101mut2.fwr** | gctgctCCCGCCCCTGCGTCTATG |
| P14 | **Q5.TSG101mut2.rvs** | agcggcTTGTACTCGATAACCTCCATGAGACG |
| P15 | **MAN343** | GTCCGTTACCCATGTTGCTGTACCAGTGGTGTTTTTGCGTCGGGTATAATAGCCATTTAATGGCGCCGTATATTTTTCAC |
| P16 | **MAN344** | GTGAAAAATATACGGCGCCATTAAATGGCTATTATACCCGACGCAAAAACACCACTGGTACAG  CAACATGGGTAACGGAC |
| P17 | **MAN345** | GCACGGACCATCCGAGGACACTACAG |
| P18 | **MAN346** | GACAAGCGAAATGGTACGAAGCGGGAC |
| P19 | **MAN347** | GGTGGATTGGTCAAGGACAGAGTTGAG |
| P20 | **MAN348** | GAAGTCGCGGAACATCTCGTTGAAGTC |
