# Supplementary material for: Toxoplasma gondii exploits the host ESCRT machinery for parasite uptake of host cytosolic proteins
Source: PLoS Pathog. 2021 Dec 13;17(12):e1010138. doi: 10.1371/journal.ppat.1010138 (PMC8700025; doi:10.1371/journal.ppat.1010138)
Supplement: S5 Table — (DOCX) [file ppat.1010138.s013.docx]

| Plasmids | Expression | Reference | Notes |
| --- | --- | --- | --- |
| pmCherry-N1-VPS4A | Vps4 wildtype fused to mCherry |  |  |
| pmCherry-N1-VPS4AEQ | Vps4 dominant negative fused to mCherry |  |  |
| pCMVNLGag-Venus | HIV-1 Gag fused to Venus | ^74^ |  |
| pCMV-Rev | HIV-1 Rev | ^75^ |  |
| pCMV-Vphu | HIV-1 Vphu gene | ^76^ |  |
| pCMVNLGagΔ*p6* | HIV-1 Gag with deletion of the p6 domain | TR^1^ |  |
| pCMVNLGagGRA14 | HIV-1 Gag expressing GRA14 predicted late domain motifs | TR^1^ |  |
| pCMVNLGagGRA14^TSG101-^ | PTAP-mutation in the GagGRA14 | TR^1^ | Substitution of the PTAP motif to AAAA |
| pCMVNLGagGRA14^ALIX-^ | YPNL mutation in the GagGRA14 | TR^1^ | Substitution of the YPNL motif to AAAA |
| pCMVNLGagGRA14^TSG101-ALIX-^ | Mutations in both of GagGRA14 late domain motifs (PTAP and YPNL) | TR^1^ | Substitution of the PTAP and YPNL motif to AAAA and AAAA |
| pYFP (pVenus) | Fluorescence reporter protein |  | Kindly provided by Dr. J. Boothroyd |
| pTRE2-mCherry | mCherry expression under the tetracycline-inducible promoter | 4 |  |
| pTet-ON | Reverse tet-responsive transcriptional activator. | 4 |  |
| pGRA14-HA | GRA14 tagged at the C-terminus with a single HA | ^26^ | Kindly provided by P. Bradley |
| pGRA14-HA^WT^_CAT | Introduction of a CAT selectable marker in the pGRA14-HA | TR^1^ |  |
| pGRA14-HA^TSG101-^_CAT | GRA14 encoding mutation in the PTAP motif | TR^1^ | Substitution of the PTAP motif to AAAA |
| pGRA14-HA^ALIX-^_CAT | GRA14 encoding mutation in the YPNL motif | TR^1^ | Substitution of the YPNL motif to AAAA |
| pGRA14-HA^TSG101-ALIX-^_CAT | GRA14 encoding mutation in the PTAP and YPNL motif | TR^1^ | Substitution of the PTAP and YPNL motif to AAAA and AAAA |

**S5 Table. Plasmids used in this manuscript**

^1^This report
