# Supplementary material for: Toxoplasma gondii exploits the host ESCRT machinery for parasite uptake of host cytosolic proteins
Source: PLoS Pathog. 2021 Dec 13;17(12):e1010138. doi: 10.1371/journal.ppat.1010138 (PMC8700025; doi:10.1371/journal.ppat.1010138)
Supplement: S6 Table — (DOCX) [file ppat.1010138.s014.docx]

| Antibodies | Dilution for  Immunofluorescence | Dilution for Immunoblot | Source |
| --- | --- | --- | --- |
| Mouse αTSG101 |  | 1:200 | Santa Cruz, Cat# sc-7964 |
| Mouse αALIX | 1:100 |  | BioRad, Cat# MCA2493 |
| Rabbit αCHMP4B | 1:200 | 1:500 | Proteintech, Cat# 13683-1-AP |
| Rabbit αPDCD6 |  | 1:2000 | Proteintech, Cat# 12303-1-AP |
| Rabbit αPEF1 |  | 1:4000 | Proteintech, Cat# 10151-1-AP |
| Mouse αMAPK7 | 1:250 |  | Origene, Cat# TA502148 |
| Rabbit αHA | 1:800 | 1:5000 | CST, Cat# 3724S |
| Rat αHA | 1:800 |  | Millipore Sigma, Cat# 118674230001 |
| Mouse αHA | 1:800 |  | BioLegend, Cat# 901533 |
| Rabbit αGRA14 | 1:500 | 1:10000 | Kindly provided by Y. Nishikawa |
| Mouse αGRA8 | 1:500 | 1:5000 | Kindly provided by G. Ward |
| Mouse αGRA1 | 1:1000 | 1:10000 |  |
| Rabbit αGRA1 | 1:2000 |  | Kindly provided by F. Spano |
| Mouse αMAG1 |  | 1:2000 |  |
| Rabbit αGRA4 |  | 1:20000 | Kindly provided by L. D. Sibley |
| Rabbit αGRA6 |  | 1:15000 | Kindly provided by L. D. Sibley |
| Rabbit GRA15 |  | 1:200 | Kindly provided by J. Saeij |
| Mouse αGRA7 | 1:1000 |  | Kindly provided by P. Bradley |
| Rabbit αGAP45 | 1:1000 |  | Kindly provided by D. Soldati-Favre |
| Rabbit αGFP | 1:200 |  | Thermo Fisher Scientific, A-11122 |
| Rabbit αTubulin |  | 1:2000 | Proteintech, Cat# 10068-1-AP |
| Human αGag |  | 1:2000 |  |

**S6 Table: Antibodies used in this study**
