# Supplementary material for: Toxoplasma gondii exploits the host ESCRT machinery for parasite uptake of host cytosolic proteins
Source: PLoS Pathog. 2021 Dec 13;17(12):e1010138. doi: 10.1371/journal.ppat.1010138 (PMC8700025; doi:10.1371/journal.ppat.1010138)
Supplement: Attachment — Submitted filename: PPATHOGENS-21-01847_response.docx [file ppat.1010138.s015.docx]

We greatly appreciate the reviewers’ feedback and their thoughtful suggestions for improving the study. Please note that references to line numbers below are for the unmarked copy.

**Reviewer #1:**

**R1C1a.** It would be nice to see some example cells for the uptake assays (e.g., Fig. 1F,G; Fig. 6). This is a rather crude assay, wouldn't it be possible to quantify the sum of material taken up per parasite?

We have included representative images from parasite uptake of host cytosolic proteins for all host-cytosolic protein uptake assays (**Fig. 1F,G, Fig. S3B, Fig. 6B,C, and Fig. 8C**). Regarding quantifying the sum of material taken up per parasite, this might seem like a relatively straightforward task, but to do it thoroughly and, ideally, in an automated manner is not trivial. We have started to develop an analysis pipeline that uses advanced software to identify parasites in phase contrast images (cellpose), recognize puncta of ingested fluorescent protein in images (resnet), and quantify intensities (cellprofiler), but the pipeline is still in development, and it will be necessary to perform new experiments and capture new images for the system to work optimally results. We expect to use the analysis pipeline in the next chapters of this work.

**R1C1b.** Can it be excluded that the reduced uptake in the GRA14KO is due to a secondary effect? This might be an alternative explanation for the lack of effect on host cell cytosol uptake when the late domain of GRA14 was mutated.

We cannot exclude that the role of TgGRA14 in the uptake of host cytosolic proteins is due through interactions with other dense granule proteins that are contributing to the recruitment of the host ESCRT machinery. We have added a statement to this effect in the discussion. (see lines 360-362).

**R1C1c.** If indeed the host ESCRT is involved in uptake into the parasite, wouldn't one expect to see TSG101 and ALIX in the parasite? Maybe in CPL-KO parasites or after LHVS treatment?

Yes, this is a possibility based on what is known about TSG101 and ALIX in relation to multivesicular body (MVB)-derived exosomes in animal cells. More specifically, TSG101 and ALIX can be packaged within the intraluminal vesicles of MVBs, which then become exosomes upon MVB fusion with the plasma membrane. As a result, TSG101 and ALIX are commonly used as markers of exosomes (Willms, E. *et al* 2016 PMID26931825). Thus, it is conceivable that these TSG101 and ALIX could similarly be internalized by *T. gondii* through the action of the host ESCRT machinery. To test this, we used our endogenously tagged GFP-TSG101 HeLa cells to assess the extent to which TSG101 is internalized and can be detected within the parasites. Initially, we performed our ingestion assay by harvesting parasites at 24hpi from GFP-TSG101 treated with 5 µM LHVS and looked for GFP-TSG101 within the parasites by microscopy. From this experiment, there was no detectable GFP-TSG101 observed in either RH DMSO-treated samples or RH LHVS samples. To enhance the sensitivity of the signal, we additionally probed with anti-GFP antibodies, but this didn’t result in visualization of the signal within the harvested parasites.

As another approached, we analyzed the presence of GFP-TSG101 within the parasites by immunoblot and, similarly to the microscopy results, we failed to detect signal derived from GFP-TSG101 in the harvested parasites. Next, we reasoned that since we can detect host derived (free, i.e., untagged) GFP within parasites by microscopy (Dou, Z. *et al* 2014 PMID25028423) it should be possible to use GFP transfected host cells as a reference. Thus, we compared expression levels of GFP in host cells transfected with a GFP plasmid versus those expressing endogenously tagged GFP-TSG101 and found that transfected host cells have approximately 10-fold higher GFP than those expressing GFP-TSG101. Thus, it is possible that GFP-TSG101 remains below the level of detection despite potentially being taken into the parasite via receptor (GRA14, and likely other parasite effectors) mediated internalization.

Because the recruitment of GFP-TSG101 at the PV is plentiful (**Fig. 4A** and **Fig. 8A**), we decided to track the recruited GFP-TSG101 by live imaging using lattice light sheet microscopy. Our results show co-localization of GFP-TSG101 with a PV luminal marker (DsRed). Due to the unexpected excess of GFP-TSG101 in what appears to be the PV lumen, it was difficult to determine if any of the GFP-TSG101 was inside the parasite. Although this is an interesting result, we will need better live cell markers to label the parasite itself rather than PV lumen to definitively evaluate internalization of GFP-TSG101, and this is not possible within the time constraints for revisions. Accordingly, we hope the reviewer agrees that it is better to address this aspect thoroughly in one of the next chapters of this work.

**R1C1d.** Conservation in apicomplexa: a host ESCRT-based mechanism for host cell cytosol uptake is not possible in malaria blood stage parasites as their host cells presumably lack ESCRT components. Nevertheless, these parasites efficiently take up host cell cytosol. Do the authors think that the ESCRT-aided mechanism of uptake is specific for T. gondii and fundamentally different to that of malaria blood stage parasites? Or is the ESCRT pathway only one of multiple pathways in T. gondii? Could it be that ESCRT has also other functions at the T. gondii PVM? This might be discussed.

The reviewer raises an interesting aspect that deserves attention. As we now cover in a new paragraph in the discussion, little is known about how hemoglobin-laden vesicles budding away from the erythrocyte cytosol and into the blood stage malaria parasites are generated. A recent study identified components of host ESCRT in mature erythrocytes and in exosome derived from reticulocytes. However, it remains unknown if erythrocyte ESCRT is functional for vesicle biogenesis. Liver stage malaria parasites presumably have the potential to exploit hepatocyte ESCRT, but the extent to which such liver stage parasites internalize cytosol from infected cells has not been reported. We convey these aspects in the discussion and state the need for additional studies to identify shared or unique mechanisms of ingestion.

**R1C2.** FigS1B: Based on the methods it can be assumed that the spinning disk microscopy-based quantification is accurate but at least in Fig. S1B it is not so obvious that the GRA14 KO has less TSG101 at the vacuole (the top panel seems to have larger cytoplasmic pool). Are these cells representative? Some explanation how to read these images might be helpful to make this difference more obvious. The same applies to Fig. S6 and Fig. 8.

The images shown reflect the natural biological variation in both the amount of TSG101 or ALIX that is expressed within host cells and the amount that is recruited to the PV. We felt it was important to select images that do not exaggerate the phenotype and instead rely on the automated quantification of recruitment based on measuring the ratio of TSG101 or ALIX occupying the area of the PV (visualized with anti-GRA1) divided by that in the host cell cytoplasm (cell perimeter, visualized by CellMask DeepRed, minus the PV and cell nucleus, visualized by Hoechst). Although this is described in the materials and methods section, we have added a brief description to the results section to clarify how recruitment was measured. Nevertheless, we recognize the importance of avoiding confusion, particularly early in the manuscript, so we also replaced the IFA images in **Fig. 2** with ones that make it easier to distinguish the phenotype.

**RIC3.** Figure 3A. Neither GRA14, ALIX, nor TSG101 really overlap very much. Is this what would be expected if GRA14 binds ALIX when viewed at SIM resolution? The data in Fig. 3B and C clearly support very close proximity and the IP data (Fig. 5A,B) strongly supports an interaction, so there seems little doubt ALIX and GRA14 and TSG101 meet. Do the authors have an explanation for the lack of full overlap in Fig. 3A?

The signal for both GFP-TSG101 and ALIX is not very bright, and this is a challenge for the low efficiency capture of light by SIM imaging. The original images were single slices from a z-stack collected by 3D SIM. To improve the signal, we have replaced the single slice images with maximum projection images. Although we recognize that this collapses the 3D space, at least this allows a better appreciation of GRA14, GFP-TSG101, and ALIX being proximal to one another. It should also be noted that interactions of these proteins are expected to be dynamic and thus one would not necessarily expect to see extensive overlap in a snapshot of time. Another potential issue is that the epitopes on GRA14-HA and ALIX could be less accessible when protein-protein interactions are engaged. The IP-MS/MS data attest to extensive and likely multicopy protein complexes within which some copies of the epitope might be accessible and other less so.

**R1C4.** Legend Figure S2 ' primer amplification sites.' Better 'primer binding site'

The suggested changes were made for **Fig. S2** legend and **Fig. S4** legend.

**R1C5.** line 277: Remove 'the' in'...within the 30 min post...'

Modified as suggested.

**R1C6.** line 341: remove one of the two 'in'

Modified as suggested.

**R1C7.** some of the references have '[Internet]' after the title, e.g. Ref11. Is this per PLosPath formatting?

Reference 11 was modified to fit *PLOS Pathogens* formatting.

**Reviewer #2:**

**R2C1.** While the GRA14 P(S/T)AP motif is conserved in Neospora, the YPXL motif does not appear to be. This should be added to the discussion. Overall, there is little support for this motif in ALIX recruitment (perhaps too close to the PTAP?). At the very least this seems to deserve adding a question mark (or three) to the model in Fig 9?

Yes, we had also noted that the YPXL motif is not conserved in NcGRA14. Although our findings from the HIV-1 VLP assays suggest that the TgGRA14 YPXL motif contributes to VLP production, there is currently no evidence of a direct interaction of the TgGRA14 YPXL motif with ALIX. Accordingly, we have separated GRA14 and ALIX in the model (**Fig. 9**) and added a question mark to reflect uncertainty in this regard.

**R2C2.** The determinants of GRA protein topology are unknown. Thus, it is a formal possibility that mutagenesis of the PTAP motif alters the topology of this protein, thereby blocking its function. The paper would be stronger if the topology of this mutant could be assessed.

Thank you for this suggestion. We performed differential permeabilization experiments to determine the topology of TgGRA14 late domain motif mutant strains and observed that in all strains the C-terminus of TgGRA14 is oriented towards the host cytosol (new **Fig. S6**). This is consistent with the topology that was described for TgGRA14 in Rome, M.E. *et al* 2008 PMID18765740. See lines 260-268.

**R2C3.** Fig. 1C. The authors should clarify how the PVM and Toxo are being detected in this figure.

The markers used to outline the PVM in **Fig.1D,E;** **Fig. S1;** **Fig. S7** and **Fig. 8A** are now included in their corresponding figure legends.

**R2C4.** Fig. 1F, G. The authors should clarify what is being scored here. It appears it is the percent of parasites with any visible venus being taken up? Are there differences in the amount of uptake in the wt vs EQ cells? It may be helpful to show a representative field for each here, particularly for this first figure.

Thank you for this suggestion, which was also made by reviewer #1. Representative images for parasites with internalized host derived cytosolic proteins are now included to clarify what is being scored in the graphs (**Fig. 1F,G**). At 30mpi (**Fig. 1F**), there is a trend toward less parasite uptake of venus in EQ cells compared to WT cells, but this potential difference did not reach statistical significance (i.e., p>0.05). However, at 24hpi significantly less parasite ingestion of venus occurs in EQ cells, suggesting a role for ESCRT in parasite uptake of host cytosolic proteins.

**R2C5.** Fig. 1G. The percentages of replicating parasites that uptake Venus is extremely low (only 6% or so, even in the ∆cpl background). It would help the readers to describe why this is expected in the text.

There are several aspects that likely explain the low apparent ingestion efficiency. First, although disruption of CPL is critical for visualizing ingested fluorescent protein (FP), our earlier work (Dou, Z. *et al* 2014 PMID25028423) showed that CPL deficient parasites still possess residual proteolytic activity in the VAC. This residual proteolysis might reduce the signal of ingested FPs to below the limit of detection in some parasites. Second, whereas host cells that are transiently transfected have high levels of FP (venus, **Fig. 1**), they represent only 30-40% of the total population because of limitations in transfection efficiency. This presumably diminishes the apparent ingestion efficiency by ~3x. Third, although cells inducibly expressing FP (mCherry, **Figs. S3, 6 and 8**) are 80% positive, the expression levels of FP in such cells are considerably lower than that of transiently transfected cells. Finally, the time at which the parasites are harvested post-infection influences ingestion efficiency, with early time points (Fig. 1F) being higher than later time points (**Figs. 1F, S3B, 6B,C, 8C**). We have added to the results section brief descriptions of how transfection efficiency and expression levels likely result in an underestimate of ingestion.

**R2C6.** Fig 2D – While the general idea that GRA14 can substitute is clear – the figure is confusing. The quantified data is from multiple experiments. The western does not particularly agree and is not described in the legend)

Quantification of western blots is intrinsically challenging because of the many steps involved in the procedure at which variation can occur, thus necessitating at least 6 experiments per condition. The legends of **Fig. 2** and **Fig. 7** were modified to include a better explanation for what it is shown in the immunoblots of the VLP assays.

**R2C7.** The term GRA14OE is vague – to what extent is this overexpressed?

This strain was kindly provided by Dr. Peter Bradley’s group and it was made by introducing a TgGRA14-HA expression construct into WT parasites (Rome, M.E. *et al* 2008 PMID18765740, which we cite in the results section). We have not quantified the extent of overexpression by western blotting, but the intensity of staining by IFA with anti-TgGRA14 is consistently higher for this strain compared to WT parasites.

**R2C8.** Figure 3A – it is difficult to visualize where the parasites are in this figure. It is also difficult to determine the extent of colocalization as claimed.

We added a DIC image to **Fig. 3A** to allow readers to better appreciate the appearance of the parasites and the position of the PV. We also used dashed lines to demarcate the PV as an additional reference point for the IFA images.

**R2C9.** Fig 3B – the author claim that the PLA assays demonstrate that GRA14 is “intimately associated” with ALIX and TSG101. There are several reports of these assays having larger than the stated 40nm distance (“intimately associated” may be an overstatement).

We have changed the wording of this sentenced was changed.

**R2C10.** The discussion of the PPYPXXXXYP motif / ALG2 comes out of nowhere. Suggest reworking this to introduce this concept better? (is this exact sequence seen or similar?)

The rationale for including the putative PPYPXXXXYP motif was further discussed. See lines 335-342. The sequence in GRA14 is PPYVPPMYP, which is a match in 5 out of 6 residues. Note that Xn can vary but is most often n=4. We now include the sequence of the motif in the results section.

**R2C11.** Fig 2C. The schematic could be made clearer with text to describe the outcome of the fusion (rather than just color coding).

The diagram for the Gagp6 domain and GRA14 C-term substitution was modified to include the specific amino acids from HIV-1 Gag that were substituted.

**R2C12.** Fig 2D – lines are not aligned well

This figure was modified to have lines aligned.

**Reviewer #3:**

**R3C1.** In order to support the recruitment of a functional ESCRT machinery at the PVM, it would be important to know whether other components of the ESCRT machinery, like ESCRT-III component CHMP4B, are also present at the PVM. In figure 1C, we would like to see CHMP4B expression (similar to panel B), instead of the PVM marker. Since the ESCRT machinery plays a vital role in membrane remodelling, the authors might also propose a role in deformation of the PVM to promote the formation of the intravacuolar network (IVN), which is known to contribute to parasite uptake (Dou et al. MBio 2014).

We have included new structure illumination microscopy (SIM) images for the recruitment of CHMP4B in the context of VPS4A^WT^ or VPS4^EQ^ (**Fig. 1D, E**). Our results showed that late acting ESCRT components such as CHMP4B and VSP4A are proximal to the PV upon disrupting ESCRT disassembly by expression of the VPS4^EQ^.

**R3C2.** We wonder if the defect in uptake of host cytosolic proteins in Δgra14 is correlated with a slight defect in growth? The growth rate of Δgra14 mutant is not analyzed in this study and was not properly documented in the previous study of Rome et al (2008) that characterized GRA14 : «the parental strain and the Δgra14 strain parasites grew with approximately the same kinetics (data not shown) ». A replication assay of Δgra14 strain would be informative for this study. Also the authors did not mention in the discussion that the defect in uptake of host cytosolic proteins in Δgra14 parasites is not due to a defect in the formation of IVN, as for GRA2 (Dou et al. MBio 2014), since no noticeable changes in IVN was observed previously in Δgra14 strain (Rome et al. Infect Immun 2008).

In new experiments we assessed the replication of RH, RΔ*gra14* and TgGRA14 late domain motif mutant strains in HeLa cells by measuring PV size and number of parasites (based on nuclear staining) per PV (now shown in **Fig. S7**). No significant differences were detected between strains, suggesting that TgGRA14 deficient strains do not exhibit a growth defect *in vitro*.

**R3C3.** The quantification shows clearly a decrease in the recruitment of GFP-TSG101 in ΔGra14 but this is not visible in the image of Figure 4A and Figure S1. Do the dots of strong intensity remain after depletion of gra14 ? Is the difference visible by eyes or only after quantification?

We have selected different images that better reflect the recruitment of GFP-TSG101 in Fig. 4. Recruitment of GFP-TSG101 can still be observed in some PVs from RΔ*gra14*, in such cases bright puncta remains at the PV.

**R3C4.** Summary : since there is no demonstration that GRA14 contributes to host cytosolic protein uptake via recruiting the ESCRT machinery, more caution should be taken in the author summary (lanes 54-55.)

We have modified Line 54 from the author summary accordingly.

**R3C5.** Fig. 1F : there is not statistic difference in the internalization of host-derived Venus between Vps4A WT and EQ mutant for newly invading parasite (only significant with the untransfected cells). The text deserves more nuancing in the conclusion (lanes 124-126). It is not surprising to see no difference at 30 min post-infection because endocytosis occurs in replicating parasite and GRAs accumulate later during intracellular replication. This result also correlates with the fact that GRA14 mutant showed normal ingestion at early time point.

We elected to cover these aspects in discussion (paragraph 2) to limit disrupting the flow of the results section.

**R3C6.** Fig. 3. Are all the vacuoles decorated with ALIX and TSG101, or only a subset? What is the % of vacuoles with an accumulation at the PVM?

Each data point in **Figs. 4B,C** and **8B** represents the ratio of PV-associated ALIX or TSG101 vs. cytoplasmic ALIX or TSG101. Since all the data points are above 1, this indicates that ALIX and TSG101 are recruited to 100% of the PVs that were analyzed. Nevertheless, the results also show considerable heterogeneity of ALIX and TSG101 recruitment to the PV, which could reflect the transient recruitment of ESCRT machinery and/or the asynchrony of the infection.

**R3C7.** Fig. 1D : the figure captions read «+Δcpl mutant » while the legend reads «infection by RH strain».

**Fig. 1** legend was modified to include <in RH or RΔ*cpl*> .

**R3C8.** Figure 4A: the figure legend does not correspond to figure 4A, but instead to figure S1. It would be much better to have figure S1 as main figure, instead of Figure 4A.

**Fig. 4** legend was modified to correspond the data illustrated in the figure.

**R3C9.** It is not clear in the text, figure and materials and methods, if the complementation of GRA14 forms are under the gra14 promoter or if they are over expression.

TgGRA14 mutations were made in the pGRA14-HA plasmid generated by D. Bradley’s group (Rome, M.E. *et al* 2008 PMID18765740). In such case, the expression of TgGRA14 is under its native promoter. This detail was included in the methods and **Fig. S5** plasmid diagram.

**R3C10.** It is not mentioned in the legend of figures which antibodies is used to stain the PVM (Figure1C, Fig. 8A ; Fig. S1, Fig. S6).

The markers used to outline the PVM in **Fig.1D,E, Fig. S1, Fig. S7 and Fig. 8A** are now included in their corresponding figure legends.

**R3C11**. Lane 123 : For readers non expert in the traffic of Toxoplasma, add a sentence to explain the reason to use Δcpl mutant or inhibitor in the experiments of host cytosolic proteins uptake.

The sentence was changed to better explain the rationale for using Δ*cpl*. See lines 126-127.

**R3C12.** Fig. 2C : the schematic for domain substitution between Gag and GRA14 is too succinct. Show the exact length and sequence of the swapping.

The diagram for the Gagp6 domain and GRA14 C-term substitution was modified to include the specific amino acids from HIV-1 Gag that were substituted.

**R3C13.** The sizes of the amplification PCR and molecular weight are missing in all figures

The molecular weight for immunoblot and PCR product sizes are now included in all figures (**Fig. 2, Fig. S2, Fig. S4, Fig. 5, Fig. 6, Fig. S5**).

**R3C14.** Line 574. The verb in the sentence is missing

This sentence was modified as requested

**R3C15.** Lines 408, 411 TgGRA14 gene is not italic.

Gene name was italicized.

**R3C16.** The authors should also mention in discussion a recent study on the role of GRA14 in the modification and control of the immune Response Mediated via the NF-κB Pathway (Ihara et al. Frontiers in Immunology 2020).

This is now included in the discussion. See line 373.
